# Engineered Protein Fibers with Reinforced Mechanical Properties Via β‐Sheet High‐Order Assembly

**DOI:** 10.1002/advs.202410199

**Published:** 2024-10-22

**Authors:** Ming Li, Dawen Qin, Jing Chen, Bo Jia, Zheng Wei, Yi Zhang, Wenhao Cheng, Qianqian Liu, Fan Wang, Jingjing Li, Hongjie Zhang, Kai Liu

**Affiliations:** ^1^ State Key Laboratory of Rare Earth Resource Utilization Changchun Institute of Applied Chemistry Chinese Academy of Sciences Changchun 130022 China; ^2^ School of Applied Chemistry and Engineering University of Science and Technology of China Hefei 230026 China; ^3^ Fuwai Hospital National Center for Cardiovascular Diseases Chinese Academy of Medical Sciences and Peking Union Medical College Beijing 100084 China; ^4^ Engineering Research Center of Advanced Rare Earth Materials (Ministry of Education) Department of Chemistry Tsinghua University Beijing 100084 China; ^5^ Xiangfu Laboratory Building 5, No.828 Zhongxing Road, Xitang Town, Jiashan Jiaxing Zhejiang 314102 China

**Keywords:** β‐sheet assembly, biological fibers, protein engineering, resilin, spidroin

## Abstract

Protein fibers are ideal alternatives to synthetic polymers due to their unique mechanical properties, biocompatibility, and sustainability. However, engineering biomimetic protein fibers with high mechanical properties remains challenging, particularly in mimicking the high molecular weight of natural proteins and regulating their complex hierarchical structures. Here, a modular design and multi‐scale assembly strategy is developed to manufacture robust protein fibers using low‐ or medium‐molecular‐weight proteins. The distinct functional and structural properties of flexible, rigid, and cross‐linked domains in modular proteins are skillfully harnessed. By regulating the ratio of rigid to flexible domains, the formation of high‐order β‐sheet crystals aligned along the fiber axis is promoted, enhancing both strength and toughness. Furthermore, the dynamic imine cross‐linking network, formed by the aldehyde‐amine condensation reaction of the cross‐linked domains, further reinforces the protein fibers. Remarkably, fibers spun from modular proteins significantly smaller than natural spidroin exhibit outstanding mechanical properties, surpassing those of protein fibers with same or even higher molecular weights. This strategy offers a promising pathway for fabricating protein fibers suitable for diverse applications.

## Graphical abstract

This study presents a modular design and multi‐scale assembly strategy for developing protein fibers with tailored mechanical properties. By adjusting the ratio of flexible and rigid domains and utilizing a dynamic imine‐bonded network, the resulting protein fibers with low‐ and medium‐molecular weights exhibit superior mechanical performance compared to protein fibers made from higher molecular weights.

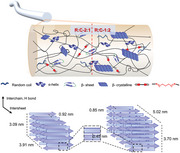

## 1 Introduction

Biomaterials in nature have long served as inspiration for the development of innovative structures, functions, and materials.^[^ ^1^, ^2^, ^3^, ^4^, ^5^, ^6^, ^7^ ^]^ In particular, protein fibers, with exceptional mechanical properties, biocompatibility, and sustainability, are attractive as compelling alternatives to synthetic polymers in various industries.^[^ ^8^, ^9^, ^10^ ^]^ Numerous structural proteins with well‐defined genetic sequences, such as elastin,^[^ ^11^ ^]^ resilin,^[^ ^12^ ^]^ keratin,^[^ ^13^ ^]^ hagfish slime thread proteins,^[^ ^14^ ^]^ and spidroins,^[^ ^15^ ^]^ have been recombinantly biosynthesized to fabricate protein fibers. However, these biomimetic protein fibers often exhibit unsatisfactory mechanical properties, limiting their applications. This shortcoming can largely be attributed to the limited molecular weight (mW) of heterologously expressed proteins and the absence of hierarchical structures in their fibers. In contrast, natural structural proteins typically possess high sequence repetitiveness and molecular weight.^[^ ^16^, ^17^, ^18^ ^]^ In addition, many natural structural protein fibers exhibit hierarchical structures, such as the nanofibrils in spider silk fibers and the spindle‐shaped microfibrils in keratin fibers.^[^ ^19^, ^20^, ^21^ ^]^ All these features are key contributors to the mechanical properties of fibers.^[^ ^22^, ^23^ ^]^ However, it remains challenging to mimic the underlying large‐mw structural proteins and their assembly and spinning processes.

Numerous efforts have been made to elevate the molecular weights of recombinant structural proteins to enhance the mechanical properties of protein fibers.^[^ ^24^, ^25^, ^26^ ^]^ For instance, high‐mW spidroins (556 kDa) were prepared by split intein‐based method, effectively boosting the mechanical performance of protein fibers to the level comparable to natural spider silk.^[^ ^27^ ^]^ However, the applications of such high‐mW engineered spidroins is restricted by low protein production, as protein yield inversely correlates with the size of the coding gene.^[^ ^27^, ^28^ ^]^ Additionally, simply increasing molecular weight may not always lead to substantial improvements, as demonstrated by man‐made megadalton titin, which yields a tensile strength of less than 400 MPa.^[^ ^29^ ^]^ Therefore, in attempts to artificially synthesize protein fibers with robust performance and high yields, engineered structural proteins have been developed beyond merely duplicating natural design. In this regard, the hybrid proteins of amyloids‐spidroins,^[^ ^30^ ^]^ squid ring teeth protein‐elastin,^[^ ^31^ ^]^ and spidroin‐elastin,^[^ ^32^ ^]^ have been designed for fabricating fibers. Nevertheless, the fused proteins have primarily been restricted to the simple proportional regulation of different modules, lacking multi‐scale assembly optimization of modular structures to achieve significant performance improvements.

In this study, we designed a series of modular proteins incorporating flexible resilin modules, rigid spidroin modules, and lysine‐rich cross‐linking modules. Incorporating additional rigid structural modules promotes crystalline domain formation and strengthens intermolecular interactions. Notably, adjusting the ratio of flexible to rigid domains effectively regulates the supramolecular interaction network mediated by intramolecular hydrogen bonds, inducing a more ordered β‐sheet structure. This approach significantly enhances the mechanical properties of modular protein fibers. In addition, a dynamic imine‐bonded cross‐linking network was established via the aldehyde‐amine condensation reaction of lysine residues, leading to both lateral and longitudinal ligation of modular proteins and increased mechanical robustness of the fibers. The modular protein with medium MW achieved fibers stronger and tougher than many high mW proteins. Thus, this multiscale regulation strategy, grounded in molecular motifs and chemical cross‐linking, provides a valuable reference for the bottom‐up development of protein fibers with customizable properties, paving the way for innovative applications in material science.

## 2 Results and Discussion

### 2.1 Design and Preparation of Multi‐Module Proteins

**Figure 1:**
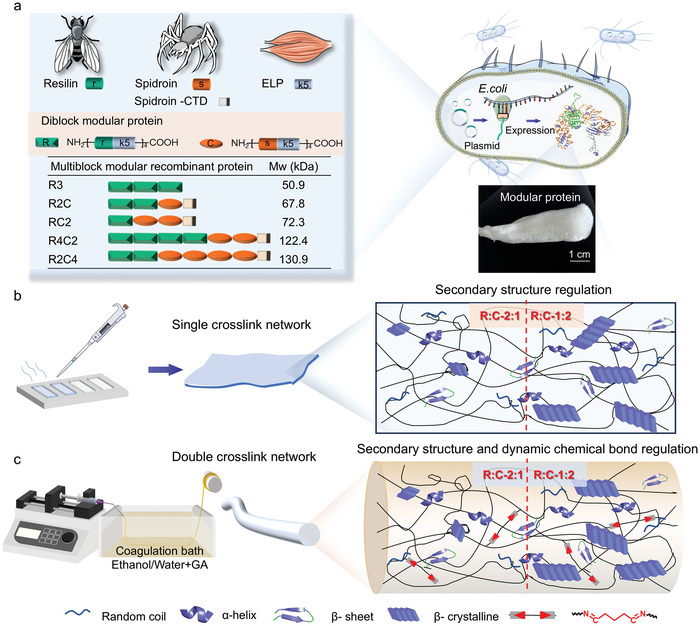
Rational design and tunable assembly of multi‐module proteins. a) Inspired by natural protein materials with excellent mechanical properties, diblock protein modules with different structures were constructed. Multi‐module protein chimeras with different block ratios and molecular weights, such as R2C, RC2, R4C2, and R2C4, were rationally designed and biosynthesized in *E. coli*. The resilin (r) motif, derived from the exon I sequence of the *Drosophila* CG15290, is an intrinsically disordered protein fragment. The spidroin (s) motif, derived from the MaSp2 variant ADF4, represents a highly folded and hydrophobic domain. The k5 motif contains lysine‐rich sequences for chemical crosslinking. The spidroin‐CTD module contributes to β‐sheet formation. Purified lyophilized multi‐module protein samples are fluffy. b) Schematic diagram of the internal structure of modular protein materials with a single crosslinked network. The mechanical properties of protein materials are fine‐tuned by modulating protein secondary structures. c) Schematic diagram of the multilevel regulation of protein material performance via protein secondary structure and dynamic amide‐bonded crosslinked network. Protein fibers were fabricated using wet spinning technology. Rapid dehydration in an ethanol/water mixture induced folded structures within the protein fibers, establishing the first physical non‐covalent crosslinking network. Subsequently, dynamic imine bonds were formed through the aldimine condensation reaction between the lysine residues of the k5 module and glutaraldehyde, creating the second chemical covalent crosslinking network.

The exceptional mechanical properties of protein materials are inseparable from their distinctive amino acid sequences and multilevel structures.^[^ ^33^, ^34^ ^]^ Inspired by the diversity of natural structural protein motifs, recombinant structural proteins can be intelligently engineered by modulating protein motifs. As illustrated in **Figure** **1**, we designed and biosynthesized multi‐module proteins by fusing flexible module R and rigid module C at various ratios. Module R consists of a diblock sequence with a 17‐amino‐acid‐long resilin‐derived sequence termed “r” (GSGGRPSDSYGAPGGGN) and an (VPGKG)_5_ sequence termed “k5”. Module C comprises a spidroin‐derived motif “s” and the “k5” motif. Specifically, the “r” motif, derived from exon I of resilin from *Drosophila* CG15920, forms a flexible random coil conformation.^[^ ^12^ ^]^ The “s” motif, derived from the fibroin 4 (ADF4) of *Araneus diadematus*, is abundant in alanine and glycine residues, forming folded structures and that serve as the strength‐providing backbone.^[^ ^35^ ^]^ The “k5” motif enables chemical cross‐linking with agents like glutaraldehyde (Table S1, Supporting Information).^[^ ^11^, ^36^ ^]^ Subsequently, modular proteins with different chimeric ratios (R2C, RC2) or larger molecular weights (R4C2, R2C4) were constructed to investigate the effect of the module ratio on protein material properties (Figure 1a). Additionally, the five‐helix C‐terminal domain (CTD) derived from spidroin has been proven critical for the aligning secondary structural features of proteins. Thus, the C‐termini of each module protein was equipped with a CTD domain derived from the natural ADF3 protein, which is crucial for fused protein to form β‐sheets structure.^[^ ^32^, ^37^ ^]^ This potential β‐sheet structures enable multi‐module proteins to non‐covalently cross‐link into macroscale materials (Figure 1b). Multi‐module proteins with varying rigid‐flexible structures ratios assemble to form crystalline domains that differ in size and content, resulting in distinct mechanical properties of protein materials. Furthermore, the imine‐bonded covalent network formed by the lysine residues of the k5 module and glutaraldehyde provides an additional mechanism to strengthen the macroscale protein material (Figure 1c). The multi‐module proteins were expressed in *E. coli*. The k5 motif contains several hydrophilic amino acids and thereby enhances soluble expression of the modular proteins. After protein expression in shake flask and purification via affinity and cation‐exchange chromatography, the production yield of modular proteins is ≈30–40 mg per liter of cell culture. The purified, lyophilized multi‐module proteins appeared fluffy. Subsequently, these multi‐module proteins were characterized by sodium dodecyl sulfate‐polyacrylamide gel electrophoresis (SDS‐PAGE), and matrix‐assisted laser desorption ionization time‐of‐flight mass spectrometry, ensuring their purity and molecular weights (Figures S1 and S2; Table S2, Supporting Information).

### 2.2 Analysis of Physically Crosslinked Low‐MW Protein Assemblies

**Figure 2:**
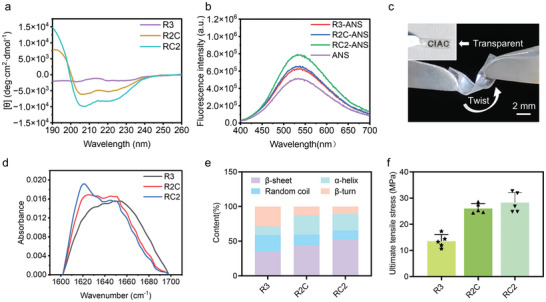
Structure and properties characterization of modular proteins and physically crosslinked protein materials. a) The CD spectra of R3, R2C, and RC2 proteins. Compared to the random conformation of R3, R2C and RC2 exhibit an α‐helix conformation with pronounced negative peaks at 208, 222 nm. b) The ANS fluorescence testing of R3, R2C, and RC2 proteins. The results indicated that the increased rigid module ratio is correlated with a significant enhancement in the protein‐ANS fluorescence signal, highlighting the transition of protein toward a more hydrophobic state. c) Images of the macroscale protein material with non‐covalently crosslinked network. The protein assembled into transparent, flexible films, allowing clear visibility of the CIAC lettering through the film. d) ATR‐FTIR spectra of R3, R2C and RC2 protein films and e) the secondary structure content analysis. The significantly enhanced peak of R2C and RC2 protein films at 1610–1640 cm^−1^ in the amide I region indicate a higher‐order β‐sheet structure. f) Mechanical properties characterization of R3, R2C and RC2 protein assemblies. The clear trend of improved mechanical performance from R3 to RC2 showcased that the variations in secondary structure directly influence the tensile strength of protein materials.

To investigate the impact of the increased rigid module C ratio on protein structure and performance, these multi‐module proteins were initially characterized by circular dichroism (CD). As expected, R3 demonstrated a random coil conformation. In contrast, R2C and RC2 exhibited an α‐helix conformation, as evidenced by distinct negative peaks at 208 and 222 nm (**Figure** **2**a). The hydrophobic probe 8‐anilinonaphthalene‐1‐sulfonic acid (ANS) was further employed to characterize the hydrophobic structure and folding aggregation state of the proteins.^[^ ^37^, ^38^ ^]^ As shown in Figure 2b, the introduction of the rigid structural domain C led to an enhanced pro‐ANS fluorescence signal, indicating that the multi‐module protein gradually aggregated and transitioned from a hydrophilic to a hydrophobic state. This observation aligns with the behavior of RC2, which exhibited limited solubility in water, revealing the influence of the rigid module C on the solubility and aggregation propensity of the multi‐module protein.

To explore the effect of secondary structure alterations induced by varying the content of module C on the mechanical performance of protein materials, protein materials with a physically crosslinked network were fabricated. Given the hydrophilicity disparities among the three modular proteins (R3, R2C, and RC2), a hexafluoroisopropanol (HFIP)/water mixed solvent was employed to produce the protein precursor solution. Of note, HFIP, a good hydrogen bond donor, enhances the secondary structure content of proteins.^[^ ^39^ ^]^ As the solvent evaporated, the precursor solution formed a transparent, bendable protein film, indicating the successful self‐assembly of multi‐module proteins (Figure 2c). Subsequently, the secondary structures of R3, R2C, and RC2 protein assemblies were investigated using attenuated total reflectance Fourier‐transform infrared spectroscopy (ATR‐FTIR). Remarkably, distinctly different intensity peaks at 1610–1640 cm^−1^ in the amide I region (1600–1700 cm^−1^) were observed for the three multi‐module proteins (Figure 2d). Further, the Fourier self‐convolutional integration and joint fitting of Gaussian and Lorentzian functions were employed to quantitatively assess the secondary structure content. The results revealed a progressive increase in β‐sheet content across the R3, R2C, and RC2 protein films, which were ≈35%, ≈44%, and ≈53%, respectively (Figure 2e; Figure S3, Supporting Information). Next, tensile testing was conducted to evaluate the mechanical properties of the three modular protein assemblies (Figure S4; Table S3, Supporting Information). Strikingly, the trends in the mechanical properties of the three protein materials were consistent with the trends in β‐sheet content. Compared to the R3 assembly, which exhibited a random structure, the RC2 protein assembly demonstrated the highest mechanical strength, nearly double that of R3 protein assembly (Figure 2f). This suggests that introducing more rigid domains leads to a tighter internal arrangement of protein molecules, facilitating protein assemblies with enhanced mechanical properties. This strategy of regulating the secondary structure to customize mechanical properties based on molecular motif regulation shows great potential for the development of high‐strength protein fibers.

### 2.3 Fabrication and Characterization of Dual‐Crosslinked Protein Fibers From Low‐MW Proteins

**Figure 3:**
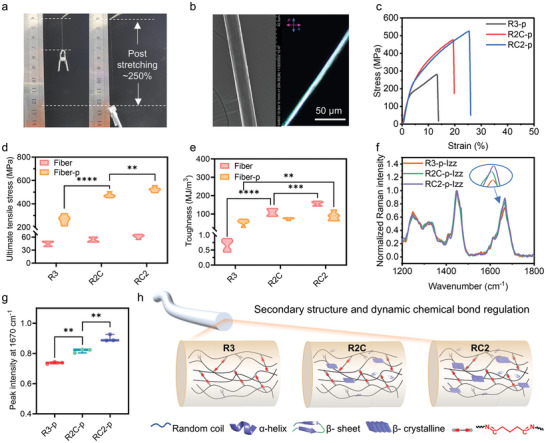
Characterization of protein fibers reinforcing by both covalently and non‐covalently cross‐linked networks. a) Multi‐module protein fibers exhibited exceptional extensibility. b) Scanning electron microscopy (SEM) (left) and polarized optical microscopy (POM) (right) analysis of the post‐stretched protein fibers, such as RC2‐p fibers. The fiber showed bright birefringence under cross‐polarized light. c) Typical stress‐strain curves of protein fibers after post‐stretching treatment. d) The mechanical strength and e) toughness of multi‐module protein fibers before and after post‐stretching treatment. f) Polarized Raman spectra of different modular protein fibers in Izz after post‐stretching treatment. g) Peak intensity of three protein fibers at 1670 cm^−1^. h) Schematic representation of the multilevel modulation of mechanical properties of protein fibers. The protein secondary structure is regulated by the rational design of protein molecular motifs. With the introduction of more folded domains, the intermolecular forces within the protein materials are enhanced, contributing to the mechanical properties. The mechanical properties of protein fibers were further modulated by a chemical crosslinking module that introduced dynamic imine crosslinking networks.

Multi‐module protein fibers were manufactured through a straightforward wet spinning strategy. The protein spinning solution was extruded into a coagulation bath containing an ethanol/water mixture with 1% glutaraldehyde, and the fibers were continuously collected on a collection roller. Ethanol could interact with various amino acids, such as glutathione, serine, glycine, and alanine, to increase intramolecular hydrogen bonds and promote the formation of ordered protein structures.^[^ ^22^, ^40^, ^41^ ^]^ In addition, ethanol induces the formation of nanoclusters and increases the size of microaggregates within proteins.^[^ ^41^ ^]^ Whereas, relying solely on the physical dehydration effect of ethanol results in a low degree of crosslinking, making the collection of aggregates difficult. Thus, it is pivotal to introduce the lysine‐rich crosslinking modules into the protein fibers. Significant improvements in mechanical properties were achieved through the chemical crosslinking network, formed by the aldimine condensation reaction between the aldehyde groups of glutaraldehyde and the amine groups of the crosslinking module k5. Both supramolecular interactions and chemical crosslinking contributed to the formation of stable protein fiber networks. Intriguingly, upon humidification, the R2C and RC2 protein fibers could be stretched to ≈250% of their original length (**Figure** **3**a). Stress‐strain curves indicated that both R2C and RC2 protein fibers exhibited excellent elongation at break. In particular, RC2 protein fibers achieved a toughness of ≈156 MJ m^−3^, with ultimate tensile strength doubling that of R3 fibers (Figure S5; Table S4, Supporting Information). This may be attributed to the abundant hydrogen bonds and increased folded structures within the RC2 protein fibers.

Post‐stretching treatment is a powerful tool for improving the mechanical properties of fibers, and the remarkable extensibility of R2C and RC2 protein fibers makes this treatment possible. Scanning electron microscopy (SEM) revealed that fibers subjected to re‐humidifying and post‐stretching exhibited smooth surfaces and uniform diameters, in stark contrast to the defected surfaces of as‐spun fibers (Figure S6, Supporting Information). Under polarized optical microscopy (POM), the fibers exhibited bright birefringent textures, indicating a well‐oriented structure within modular protein fibers (Figure 3b). Additionally, sharp textures on the equator, observed in synchrotron radiation small‐angle X‐ray scattering (SR‐SAXS), confirmed the highly ordered molecules arrangement within post‐stretched protein fibers (Figure S7, Supporting Information). FTIR analysis further investigated the secondary structure variability of multi‐module protein fibers before and after post‐stretching treatment. Quantitative analysis using Fourier convolution revealed an increase in β‐sheet structure content after the post‐stretching treatment (Figure S8, Supporting Information). In view of this, we speculate that during the initial re‐humidification, the plasticization of water causes the fibers to expand and curl, additional hydrogen bonds to form inside the molecules, destroying some original dynamic imine bonds.^[^ ^11^ ^]^ In addition, water molecules accelerate the molecular chain movement. The external force applied during stretching promoted the internal rearrangement of the molecules, with random segments becoming highly oriented, contributing to the formation of the folded structure. Notably, dynamic imine bonds reorganized during dehydration,^[^ ^11^ ^]^ laying the foundation for the high performance of multi‐module protein fibers (Figure S9, Supporting Information).

Next, the mechanical properties of post‐stretched protein fibers (R3‐p, R2C‐p and RC2‐p fibers) were evaluated using tensile testing. Representative stress‐strain curves are shown in Figure 3c. The mechanical properties of the multi‐module protein fibers were significantly improved after post‐stretching, albeit with some reduction in toughness (Figures 3d,e; Figure S5; Table S4, Supporting Information). Notably, the mechanical properties of R2C‐p fibers increased nearly ninefold compared to R2C fibers, up to ≈473 MPa. Overall, as the proportion of the rigid module C increased, there was a consistent trend of improvement in both the strength and toughness of the fibers. Particularly, RC2‐p fibers achieved a performance as high as 523 MPa, comparable to that of most recombinant protein fibers.^[^ ^29^ ^]^ This enhancement is primarily attributed to the crucial reinforcing role of the β‐sheet structure in structural protein fibers.

Further, the microstructure of multi‐module protein fibers was investigated by polarized Raman spectroscopy. The polarization signals of the three multi‐module protein fibers were collected parallel (zz direction) and perpendicular (xx direction) to the fiber axis.^[^ ^29^, ^42^ ^]^ As shown in Figure 3f,g, the peak intensities of R3‐p, R2C‐p, and RC2‐p fibers at 1670 cm^−1^ in the amide I band exhibited a significant increasing trend after normalization at 1447 cm^−1^. The results indicate that increasing the ratio of the rigid module C contributes to higher intensities of crystalline domains along the fiber axis. Additionally, the unique dimerization site provided by CTD domain has been reported to induce the assembly of protein aggregates to form β‐sheet structures arranged orderly along the long axis of the fiber,^[^ ^32^, ^37^ ^]^ which synergistically enhances the 1670 cm^−1^ peak intensities on the Izz direction. In contrast, there was no obvious change for the peak at the perpendicular direction (Figure S10, Supporting Information). Collectively, RC2‐p fibers possess more highly oriented and denser β‐sheet structures along the fiber axis. Based on this, we propose a mechanism for designing multi‐module protein fibers with customizable properties (Figure 3h). At the macroscopic scales, the dynamic imine‐bonded crosslinked network, formed through the aldimine condensation reaction between the k5 crosslinking module and glutaraldehyde, underpins the high mechanical properties of the fibers. Post‐stretching treatments effectively enhance intramolecular orientation and promote the formation of protein secondary structures. Additionally, varying the ratio of the rigid module C results in different intensities of crystalline domains along the fiber axis. Increasing the module C content boosts crystallization signals and strengthens intermolecular forces, resulting in improved mechanical properties of protein fibers. Therefore, by regulating the β‐sheet domain content and establishing a reasonable dynamic crosslinking network, the performance of protein fibers can be precisely tailored.

### 2.4 Structural and Mechanical Characterization of Medium‐MW Multi‐Module Protein Fibers

**Figure 4:**
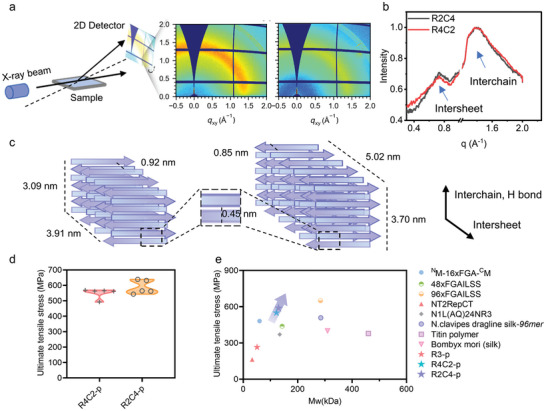
Characterization of the structure and mechanical properties of fibers spun from medium‐MW multi‐module proteins. a) 2D GIWAXS patterns for R4C2 (left) and R2C4 (right). b) Structural analysis of R4C2 and R2C4. The two detected peaks represent the inter‐chain and inter‐sheet distances, respectively. c) Schematic crystal structures of R4C2 (left) and R2C4 (right) based on GIWAXS analysis. For R4C2, the average crystallite size was 3.91 nm, indicating 4–5 β‐sheets per crystal. Whereas, the average crystallite size for R2C4 was 5.02 nm, corresponding to 5–6 β‐sheets per crystal. d) Mechanical properties of R4C2‐p and R2C4‐p fibers. e) Stress‐MW plot of various natural and synthetic protein‐based fibers.

Inspired by natural spider silk, higher MW of protein is closely associated with stronger tensile properties. Given the outstanding performance of multi‐module structural proteins in modulating the properties of protein fibers, medium‐MW proteins with the same chimera ratio were further synthesized and characterized. The structure of the R4C2 and R2C4 protein films on silicon was first determined by grazing‐incident wide‐angle X‐ray scattering (GIWAXS) (**Figure** **4**a). The 2D diffraction patterns of multi‐module proteins showed signals for all half‐rings, indicating isotropic structures resulting from the drop‐casting method. The results highlighted two different reflections, correlating with inter‐sheet and inter‐chain distances, respectively (Figure 4b). The d‐spacings for the two crystallization peaks of the R4C2 protein were 0.45 and 0.92 nm, while those for the R2C4 protein were 0.45 and 0.85 nm, respectively. Of note, the inter‐chain d‐spacing of both modular proteins was 0.45 nm, consistent with natural spider silk.^[^ ^30^ ^]^ While the inter‐sheet distances were higher than those in natural silk, which might be mainly attributed to the strong positive charge mutual repulsion in the ring region of the β‐sheet structure of the muti‐module protein.^[^ ^30^, ^43^ ^]^ Specifically, the higher randomness in the R4C2 muti‐module protein allowed for greater movement of the positively charged protein chain, resulting in a slightly larger inter‐sheet distance compared to the R2C4 protein. The average crystallite sizes were further assessed by the Debye‐Scherrer equation.^[^ ^29^ ^]^ For R4C2 muti‐module protein, the average crystallite sizes along the inter‐sheet axis and inter‐chain axis were 3.91 and 3.09 nm, respectively, indicating the presence of 4–5 sheets, each with 6–7 β‐strands. In contrast, the R2C4 muti‐module protein displayed larger average crystallite sizes of 5.02 nm along the inter‐sheet axis and 3.70 nm along the inter‐chain axis (Figure 4c). This disparity in average crystallite sizes and intermolecular forces significantly contributes to the divergence in their mechanical properties.

The structure and mechanical properties of medium‐MW multi‐module protein fibers were further explored. Benefiting from the post‐stretching treatment, the surface of the protein fibers was smooth (Figure S11, Supporting Information). The POM and SAXS confirmed the exceptional structure orientation of protein fibers (Figure S12, Supporting Information). Moreover, FTIR analysis demonstrated that the post‐stretched protein fibers behaved with more β‐sheet, echoing the trend observed in fibers made from lower MW proteins (Figure S13, Supporting Information). Notably, the β‐sheet content in R2C4‐p fibers surpassed that in R4C2‐p fibers, aligning with the previous GIWAXS results that showed more crystalline domains in R2C4 protein. Indubitably, R2C4‐p fibers displayed superior mechanical performance compared to R4C2‐p fibers, with an ultimate tensile strength of 588.25 ± 39.30 MPa, toughness of 90.60 ± 13.98 MJ m^−3^, and a modulus of 7.49 ± 0.33 GPa (Figure 4d; Figure S14; Table S5, Supporting Information). This is primarily attributed to the denser packing of the internal β‐sheet, stronger intermolecular forces, and larger average crystallite sizes within the protein molecules. Moreover, the mechanical properties of medium‐MW protein fibers are significantly higher than those of low‐MW ones at the same domain ratio (Figure S15, Supporting Information). This indicates that more interchain interactions as well as fewer chain ends contribute to the mechanical properties of the protein fibers. Significantly, the ultimate tensile strength of R2C4‐p fibers exceeds that of many reported protein fibers with the equivalent or even higher MW (Figure 4e).^[^ ^29^, ^30^, ^44^, ^45^, ^46^, ^47^ ^]^

Additionally, the biocompatibility of these fibers was assessed by co‐culturing them with various cell lines, including HEK293T, L929 and HT22 cells. Utilizing calcein‐AM and propidium iodide (PI) dual staining to differentiate live and dead cells, it was observed that the protein fibers exhibited negligible toxicity. Moreover, cells adhered well to the fiber surface and proliferated effectively (Figure S16, Supporting Information), likely due to the complex interactions between the fibers and cells. The fibers may provide a reaction force that regulates cell behavior,^[^ ^48^ ^]^ or establish hydrophobic interactions with cells,^[^ ^49^ ^]^ contributing to enhanced cell proliferation and adhesion. Consequently, these multi‐module protein fibers hold immense promise for applications in tissue engineering, due to their biocompatibility and tailored mechanical performance.

## 3 Conclusion

By combining the structural and physicochemical properties of different protein modules, we propose a modular design and multi‐scale assembly strategy for the development of protein fibers with reinforced mechanical properties. The protein microstructures can be precisely manipulated by regulating the ratio of rigid to flexible modules. The increasing of rigid domain proportion contributes to more ordered and dense crystallite packing, enhancing the intermolecular interactions among protein chains. In addition, the dynamic imine bonds between lysine residues in the cross‐linking module further connected protein chains and contribute to the robust mechanical properties. Remarkably, fibers spun from modular protein with medium molecular weight displayed impressive mechanical properties, surpassing many recombinant protein fibers of the same or even higher MW categories. Moreover, the biocompatibility of these modular protein fibers enables their potential for biomedical applications. Overall, this modular protein design and assembly strategy paves the way for the development of novel protein materials with customizable properties, offering vast potential across various fields.

## Conflict of Interest

The authors declare no conflict of interest.

## Supporting information



Supporting Information

## Data Availability

The data that support the findings of this study are available from the corresponding author upon reasonable request.
