# Supplementary material for: Engineered Protein Fibers with Reinforced Mechanical Properties Via β‐Sheet High‐Order Assembly
Source: Adv Sci (Weinh). 2024 Oct 22;11(46):2410199. doi: 10.1002/advs.202410199 (PMC11633540; doi:10.1002/advs.202410199)
Supplement: Supplementary file 1 — Supporting Information [file ADVS-11-2410199-s001.docx]

Supporting Information

Engineered protein fibers with reinforced mechanical properties via β-sheet high-order assembly

## Materials and Methods

## Materials

The DNA sequences encoding the R-C unit were synthesized from GENEWIZ (Suzhou, China). The restriction enzymes were purchased from TransGen (China). *E. coli* BLR (DE3) competent cells for protein expression were bought from Novagen (USA). Ni-NTA columns, SP HP columns and Desalting columns for protein purification were purchased from General Electric Company (USA). All biochemicals for protein expression, such as LB medium, salts, antibiotics as well as inducer compounds, were used as received from Sigma-Aldrich. Hexafluoroisopropanol (HFIP) was provided by Bide Pharmatech Ltd. Glutaraldehyde was supplied by Energy Chemical. The ultrapure water used in this research (18.2 MΩ cm) was from a Milli-Q ultrapure water system (Merck, Germany).

## Plasmid construction

The synthetic gene element “*Nde*I-*PflM*I-[r-k5]_4_-*Bgl*I-*Nhe*I-[s-k5]4-*Spe*I-His6-*EcoR*I” was integrated on the m13 vector. Fragment R ([r-k5]_4_) or fragment C ([s-k5]_4_) was obtained by double digestion with *PflM*I and *Bgl*I or double digestion with *Nhe*I and *Spe*I. Utilizing T4 DNA ligase, these fragments were then ligated to the linearized m13 vector, either with a single *PflM*I or *Nhe*I restriction, yielding the intermediate vectors R2C-m13 and RC2-m13, respectively. Subsequently, the spidroin-CTD fragment, obtained from the existing plasmid in the laboratory,^[1]^ was connected to the C-terminus of the intermediate vector. After verifying the correct connection direction by DNA sequencing, the modular protein vectors R2C-m13, RC2-m13 were obtained and preserved for later use.

Furthermore, the fragment C2-CTD digested from RC2-m13 was connected to R2C-m13, and both fragments were treated with *Nhe*I and *EcoR*I, resulting in the vector R2C2-m13. Based on this, we digested R2C2-m13 with *Nhe*I and *EcoR*I to obtain the C2-CTD fragment, which was connected to the R2C2-m13 vector linearized with *Spe*I and *EcoR*I, culminating in the production of R2C4-m13. Similarly, the C2-CTD fragment was ligated to the previously developed plasmid RS16,^[2]^ yielding the R4C2-m13 vector. To express modular proteins in *E. coli*, the multimodule protein genes R2C, RC2, and R2C4 were ligated into the expression vector pET-25b through digestion with *Nde*I, *EcoR*I and ligation with T4 DNA Ligase. Each step of digestion and connection was followed by colony PCR to identify positive colonies and subsequently confirmed by DNA sequencing.

## Protein expression and purification

The constructed plasmids containing target genes were transformed into chemically competent *E. coli* BLR (DE3) and then plated on LB agar plates. A single clone was inoculated in 100 mL of LB medium containing 100 µg·mL^-1^ ampicillin, and incubated at 37℃ for 7-8 h. Subsequently, 15 mL of the seed culture was transferred into a 5 L shake flask containing 1 L of TB medium, incubated at 37℃, 220 rpm. When the cell OD600 reached 0.6-0.8, isopropyl β-D-1-thiogalactopyranoside (IPTG) was added in a final concentration of 0.1 mM and the culture temperature was downshifted to 28.5℃. After shaking overnight, the cells were harvested by centrifugation and stored at -80℃.

The cells were resuspended in a lysis buffer (50 mmol∙L^-1^ sodium phosphate buffer, 300 mmol∙L^-1^ NaCl, 20 mmol∙L^-1^ imidazole, pH 8.0), disrupted using a cell ultrasonic disruptor, and the supernatant was collected by centrifugation. Proteins were purified from the supernatant under ambient conditions using Ni Sepharose 6×FF column. The protein-containing fractions were dialyzed to remove excess salt and then purified using an SP HP column. Additionally, the elution products were purified by a desalting column. Purified proteins were frozen, lyophilized, and stored at -80 ℃ for further use.

## SDS-PAGE analysis

The purity of the protein was detected using a 12% sodium dodecyl sulfate polyacrylamide gel electrophoresis (SDS-PAGE).

## Matrix-Assisted Laser Desorption/Ionization Time-of-Flight Mass Spectrometry analysis

The protein samples were dissolved in ultrapure water at 1 mg·mL^-1^ for mass spectrometric analysis (autoflexIII MALDI-TOF Bruker, German).

## Circular Dichroism Spectrum (CD) analysis

CD spectra were acquired using a Jasco J-820 CD spectrometer. Proteins were diluted in hexafluoroisopropanol/water (4/1, v/v) solvents to a concentration of 0.2-0.5 mg·mL^-1^. The CD spectra were obtained at 20 ℃, scanning in 0.5 nm steps from 190-260 nm with a 1 nm bandwidth, and a 2 s response time. Multiple spectra were acquired, each being the average of triplicate scans.

## Fluorescent spectrometry analysis

Spectra were recorded by an F-7000 fluorescence spectrophotometer. 40 μM ANS was added to proteins dissolved in hexafluoroisopropanol/water solvents, and incubated for 2-3 h at room temperature. Subsequently, the fluorescence emission between 400 and 700 nm was monitored at an excitation wavelength of 355 nm.

## Preparation of the single crosslinked network protein materials

Multimodule protein was dissolved in hexafluoroisopropanol/water (4/1, v/v) at a concentration of 160 mg·mL^-1^. Then transfer the precursor solution to a mold with dimensions of 15 mm in length, 5 mm in width, and 1 mm in thickness. Transparent films were formed upon the solvent evaporates.

## Fourier transform infrared (FTIR) analysis

Spectra of the samples were acquired from 400-4000 cm^-1^ at 4 cm^-1^ resolution using a Germany Bruker INVENIO-R FTIR spectrometer.

## Preparation of the double crosslinked network protein materials

The double crosslinked network protein fibers were prepared via wet spinning technology. In brief, the protein precursor solution was extruded into a coagulation bath of ethanol/water (95/5, v/v) and 1% GA to form fibers, which were collected on a collection roller.

## Tensile test

The tensile test of different multimodule protein materials was acquired on the FAVIMAT+ instrument (Textechno, German, 12 N). The single crosslinking network protein materials was performed in a gauge length 10 mm and a speed of 5 mm·min^-1^. The protein fiber was measured in a gauge length 4 mm and a speed of 5 mm·min^-1^.

## Scanning Electron Microscopy (SEM) analysis

Morphology observation was performed using an S-4800 scanning electron microscopy (SEM, Hitachi, Japan) at 10 kV. The samples were mounted onto the specimen stubs by means of conductive double-sided adhesive tape and sputtered with gold for 30 s.

## Polarized optical microscopy (POM) analysis

The molecular orientation and birefringence of multimodule protein fiber before and after post-stretched treatment was examined by a polarized optical microscope (Nikon, ECLIPSE LV100N).

## Polarization Raman analysis

Polarized Raman spectra were obtained using a HR Evolution (HORIBA Jobin Yvon, Japan). Specifically, fiber samples were oriented along the x-axis and were irradiated using a 532 nm line argon laser with polarization fixed along the x-axis. Subsequently, the fibers were realigned to the y-axis, and the data acquisition process was repeated. The Raman data were analyzed with Labspec 6 software. All spectra were normalized to the intensity of the 1447 cm^-1^ peaks, which arises from CH_3_ asymmetric stretching and CH_2_ bending and is insensitive to protein conformation.

## Synchrotron Radiation Small Angle X-ray Scattering (SR-SAXS) analysis

Synchrotron Radiation Small Angle X-ray Scattering (SR-SAXS) analysis of fibers was carried out in the BL19U2 station of Shanghai Synchrotron Radiation Facility (SSRF). The chosen X-ray wavelength (λ) was 0.889 nm (energy was 13.95 keV). The sample-to-detector distance was 2753 mm. The exposure time for each fiber measurement ranged from 5 s. SAXS analysis was carried out by Fit 2D.

## Grazing-Incident Wide-Angle X-Ray Scattering (GIWAXS) analysis

Protein solution was drop-coated onto bare silicon substrate. Grazing-incidence wide-angle X-ray scattering (GIWAXS) analysis of protein samples was carried out on beamline 1W1A (λ = 1.54 Å) at the Beijing Synchrotron Radiation Facility (BSRF). The incidence angle was 0.2°, the distance of the sample to CCD was 284 mm and the exposure time was 100 s. The peak at a smaller q corresponds to the distance between β-sheets while the peak at larger q corresponds to the interchain distance.^[3]^

## Biocompatibility of the protein fibers

Diverse cell lines were employed to evaluate the cytotoxicity of the fibers. Human embryonic kidney cells 293T and Mouse hippocampal neuronal cells HT22 were cultured in DMEM medium supplemented with 10% FBS, while mouse fibroblast cells L929 were cultured in 1640 medium supplemented with 10% FBS. All cells were seeded at a density of 10^5^ cells per well in a 12-well plate and co-cultured with UV-sterilized fibers for 24 h. 1 µL of calcein-AM staining and 1 µL of propidium iodide (PI) were added to each well and incubated for 20 min. Subsequently, dyes were removed and washed with PBS buffer, and images of cells with fiber were photographed by LSCM at an emission wavelength of 488 nm for AM and 561 nm for PI.

## Statistical analysis

Origin 2023 and GraphPad Prism 9 software were used for statistical analysis of data. All results were shown as mean ± standard deviation (SD). One-way or two-way analysis of variance (ANOVA) was used to generate p-value to determine statistical significance. (*p < 0.05, **p < 0.01, ***p < 0.001, ****p < 0.0001).

## Supplementary Figures and Tables

**Table S1.** Amino acid sequences of protein modules used in this work.

| Protein modules | Sequences |
| --- | --- |
| R | GSGGRPSDSYGAPGGGN |
| S | GSSAAAAAAAASGPGGYGPENQGPSGPGGYGPGGP |
| Spidroin-CTD | GAASAAVSVGGYGPQSSSAPVASAAASRLSSPAASSRVSSAVSSLVSSGPTNQAALSNTISSVVSQVSASNPGLSGCDVLVQALLEVVSALVSILGSSSIGQINYGASAQYTQMVGQSVAQALAG |
| k5 | [VPGKG]_5_ |
| R | [VGSGGRPSDSYGAPGGGNP(VPGKG)_5_VPG]_4_ |
| C | [GSSAAAAAAAASGPGGYGPENQGPSGPGGYGPGGP(VPGKG)_5_TS]_4_ |


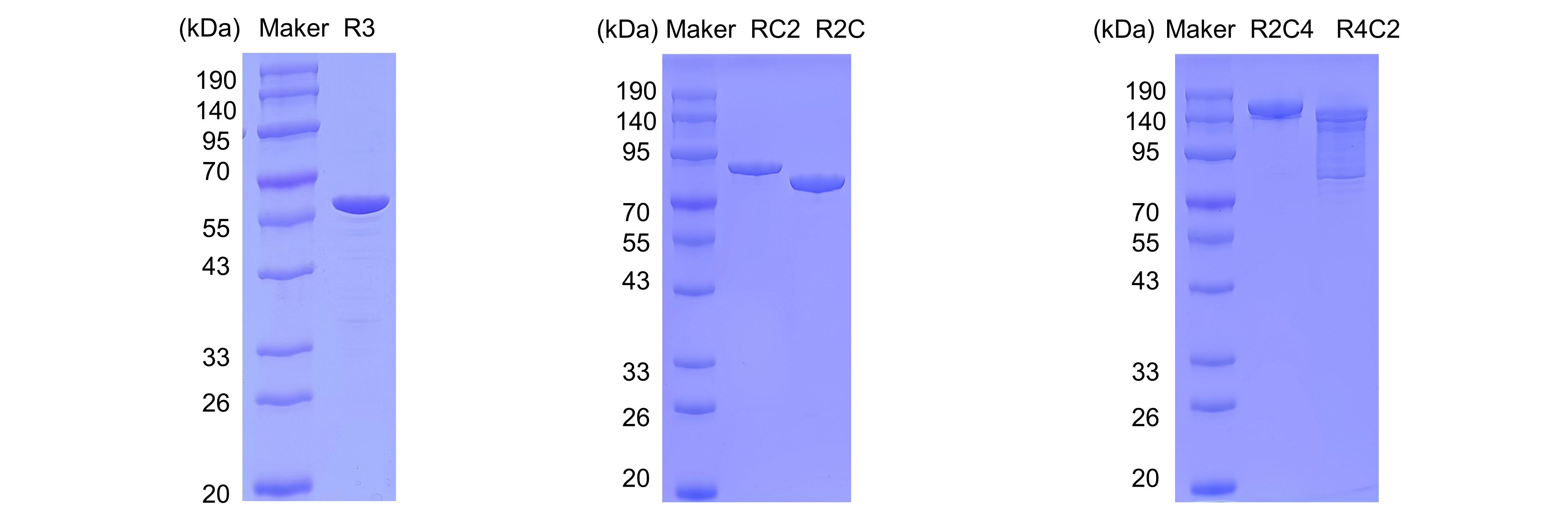


**Figure S1.** The SDS-PAGE characterization of purified multimodule proteins used in this study.


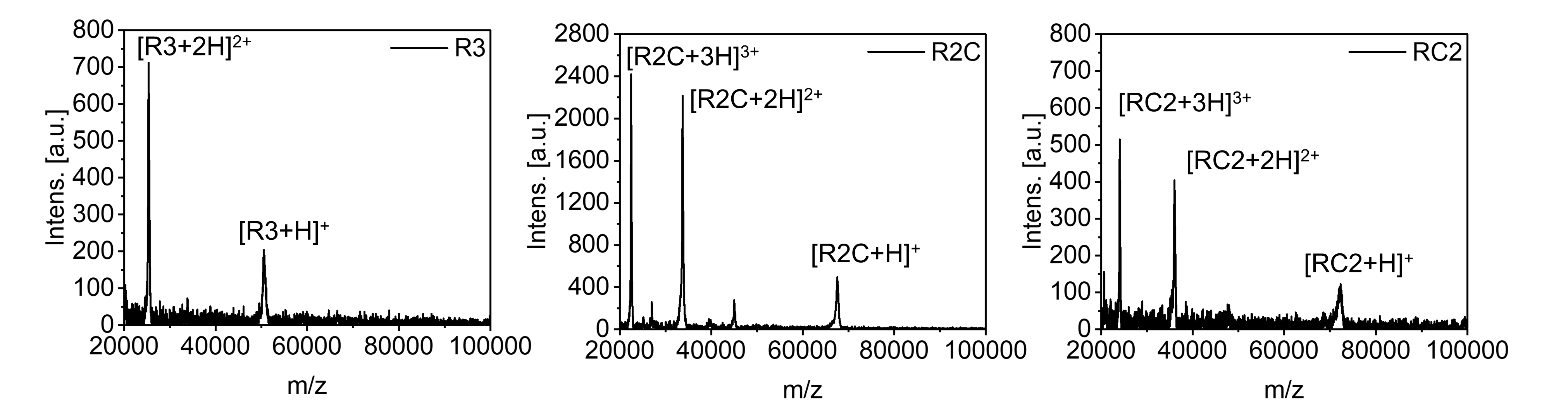


**Figure S2.** Matrix-assisted laser desorption/ionization time-of-flight (MALDI-TOF) mass spectra of the multi-module proteins (R3, R2C, RC2). The molecular weight values determined by mass spectrometry were in good agreement with the theoretical Mw (shown in Table S2).

**Table S2.** General information of proteins used in this work.

*: Molar mass calculated with ProtParam (<https://web.expasy.org/protparam/>).

^#^: Molar mass determined by MALDI-TOF mass spectrometry.

| Modular protein name | Sequence | *M*_calculated_(Da)* | *M*_MS_(Da)^#^ |
| --- | --- | --- | --- |
| R3 | MGQG[VGSGGRPSDSYGAPGGGNP(VPGKG)_5_VPG]_12_WHHHHHH | 50801.0 | 50520.9 |
| R2C | MTQG[VGSGGRPSDSYGAPGGGNP(VPGKG)_5_VPG]_8_WHGAS[GSSAAAAAAAASGPGGYGPENQGPSGPGGYGPGGP(VPGKG)_5_TS]_4_GAASAAVSVGGYGPQSSSAPVASAAASRLSSPAASSRVSSAVSSLVSSGPTNQAALSNTISSVVSQVSASNPGLSGCDVLVQALLEVVSALVSILGSSSIGQINYGASAQYTQMVGQSVAQALAGTSHHHHHH | 67766.5 | 67582.4 |
| RC2 | MTQG[VGSGGRPSDSYGAPGGGNP(VPGKG)_5_VPG]_4_WHGAS[GSSAAAAAAAASGPGGYGPENQGPSGPGGYGPGGP(VPGKG)_5_TS]_8_GAASAAVSVGGYGPQSSSAPVASAAASRLSSPAASSRVSSAVSSLVSSGPTNQAALSNTISSVVSQVSASNPGLSGCDVLVQALLEVVSALVSILGSSSIGQINYGASAQYTQMVGQSVAQALAGTSHHHHHH | 72351.1 | 72317.0 |


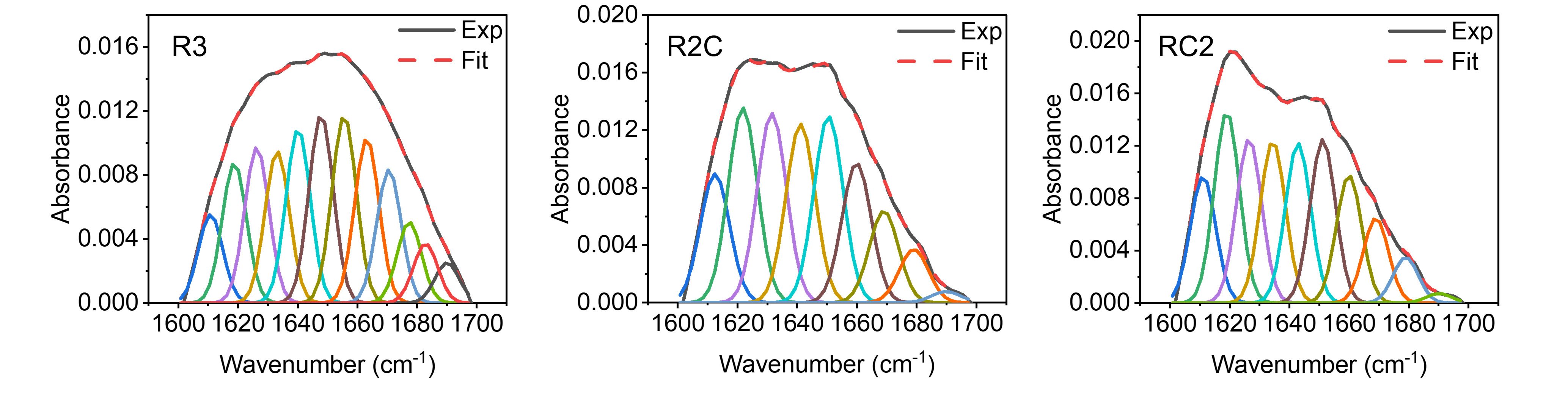


**Figure S3.** Peak fitting in FTIR amide I region of the single crosslinked network protein materials.


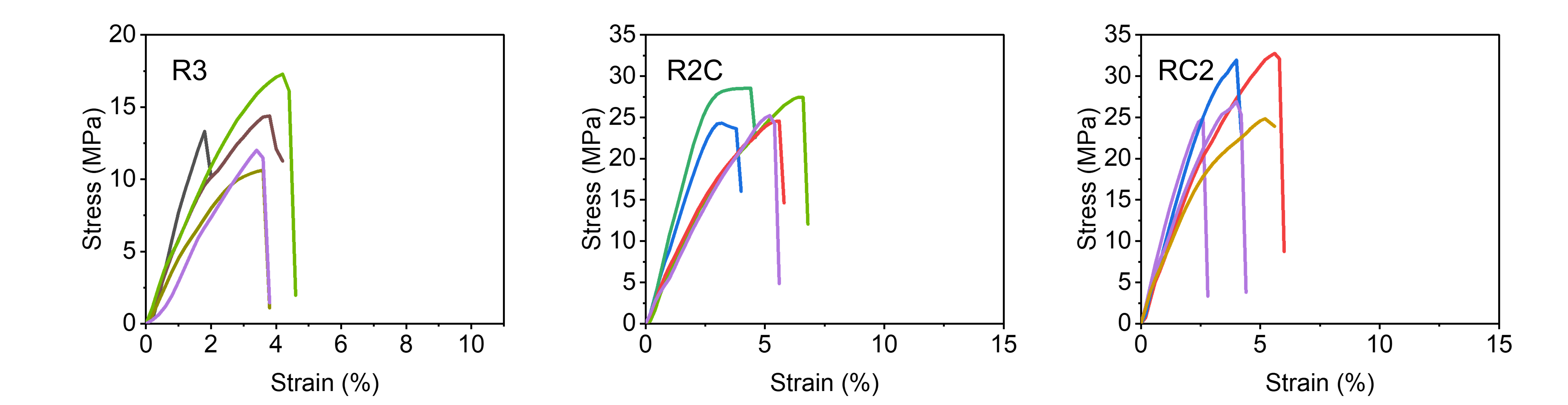


**Figure S4**. Typical stress-strain curves of the physically crosslinked protein materials.

**Table S3**. Summary of mechanical properties of the physically crosslinked protein materials in this work. All data are presented as mean ± SD (n = 5).

|  | Stress (MPa) | Toughness (MJ/m^3^) | Modulus (GPa) |
| --- | --- | --- | --- |
| R3 | 13.53±2.87 | 0.30±0.12 | 0.53±0.06 |
| R2C | 26.02±1.68 | 0.88±0.17 | 0.76±0.21 |
| RC2 | 28.27±3.43 | 0.80±0.26 | 0.87±0.12 |


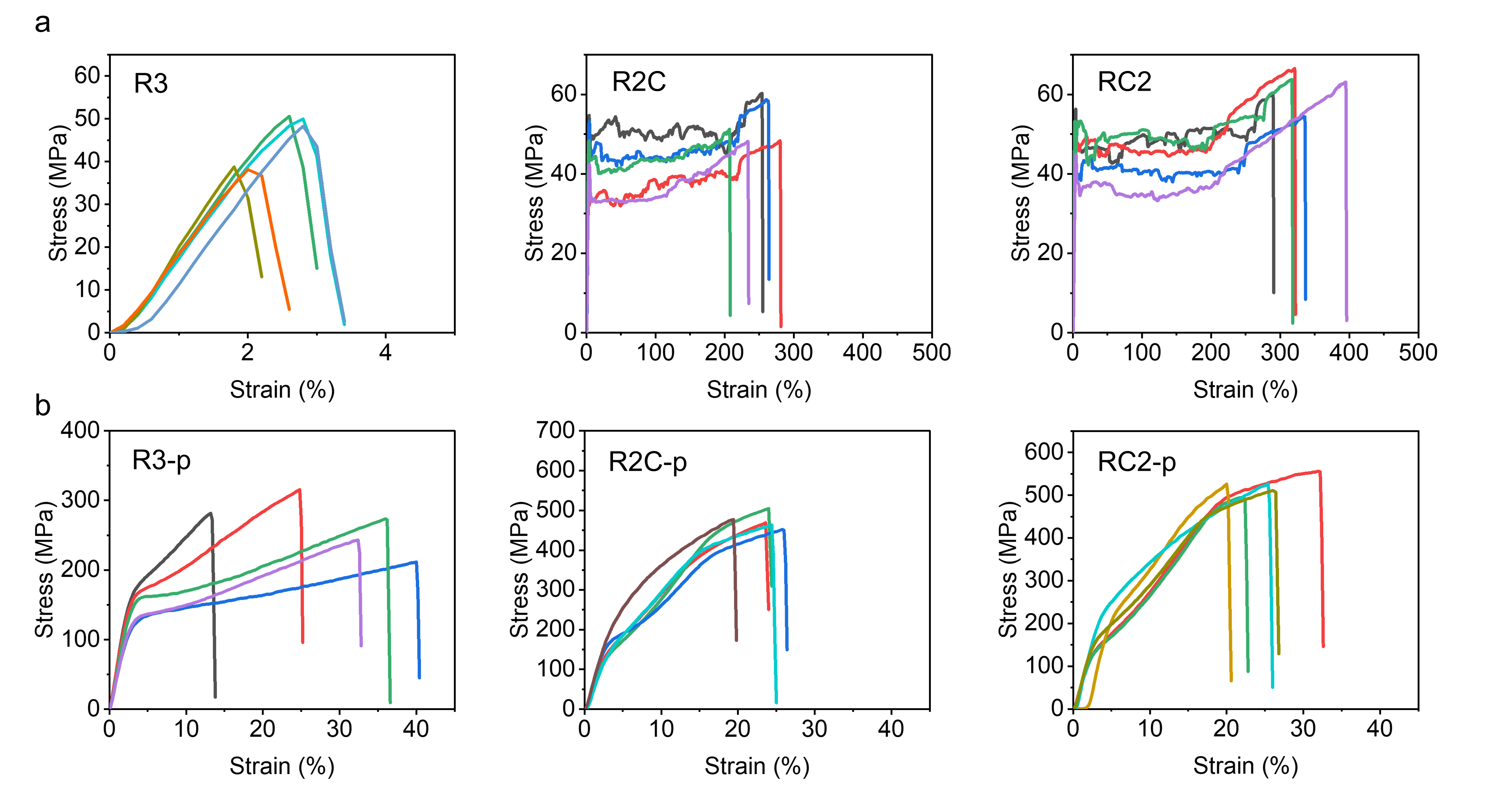


**Figure S5**. Typical stress-strain curves of R3, R2C, RC2 protein fibers before (a) and after (b) post-stretching.

**Table S4**. Summary of mechanical properties of as-spun R3, R2C, RC2 protein fibers and post-stretched R3-p, R2C-p, RC2-p protein fibers in this work. All data are presented as mean ± SD (n = 5).

|  | Diameter (μm） | Stress  (MPa) | Toughness (MJ/m^3^) | Modulus (GPa) | Fracture strain (%) |
| --- | --- | --- | --- | --- | --- |
| R3 | 15.83±0.96 | 45.15±5.52 | 0.68±0.17 | 2.30±0.15 | 2.4±0.42 |
| R2C | 29.93±3.82 | 53.35±5.15 | 108.12±16.78 | 1.83±0.24 | 247.02±24.94 |
| RC2 | 32.72±4.96 | 61.54±4.15 | 156.77±10.64 | 2.05±0.22 | 331.52±34.91 |
| R3-p | 6.73±0.58 | 265.12±35.35 | 55.11±15.35 | 5.40±0.78 | 29.28±9.47 |
| R2C-p | 10.52±1.75 | 473.25±17.68 | 74.05±5.45 | 6.02±0.50 | 23.44±2.15 |
| RC2-p | 11.16±3.09 | 523.08±20.06 | 87.05±21.11 | 6.52±1.06 | 25.12±4.07 |


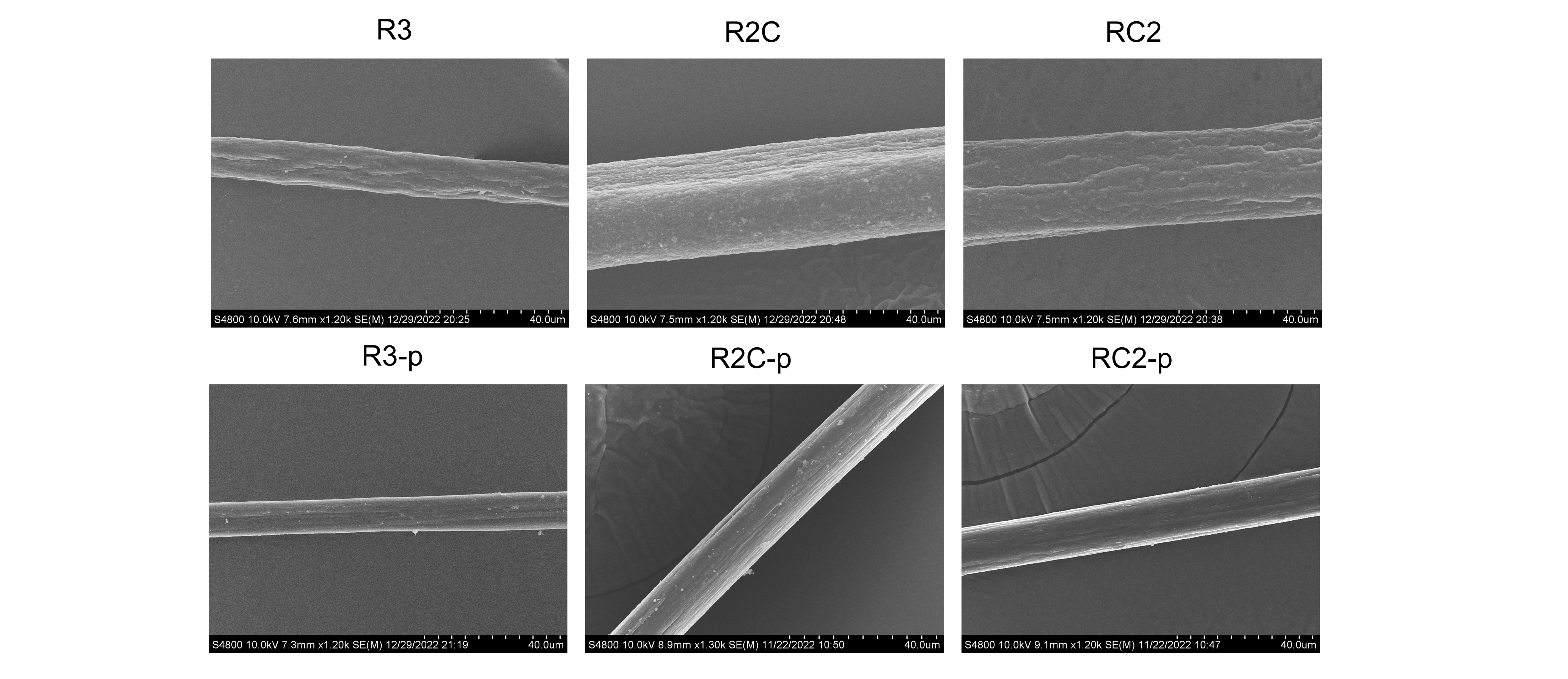


**Figure S6.** SEM images of as-spun R3, R2C, RC2 protein fibers and post-stretched R3-p, R2C-p, RC2-p fibers.


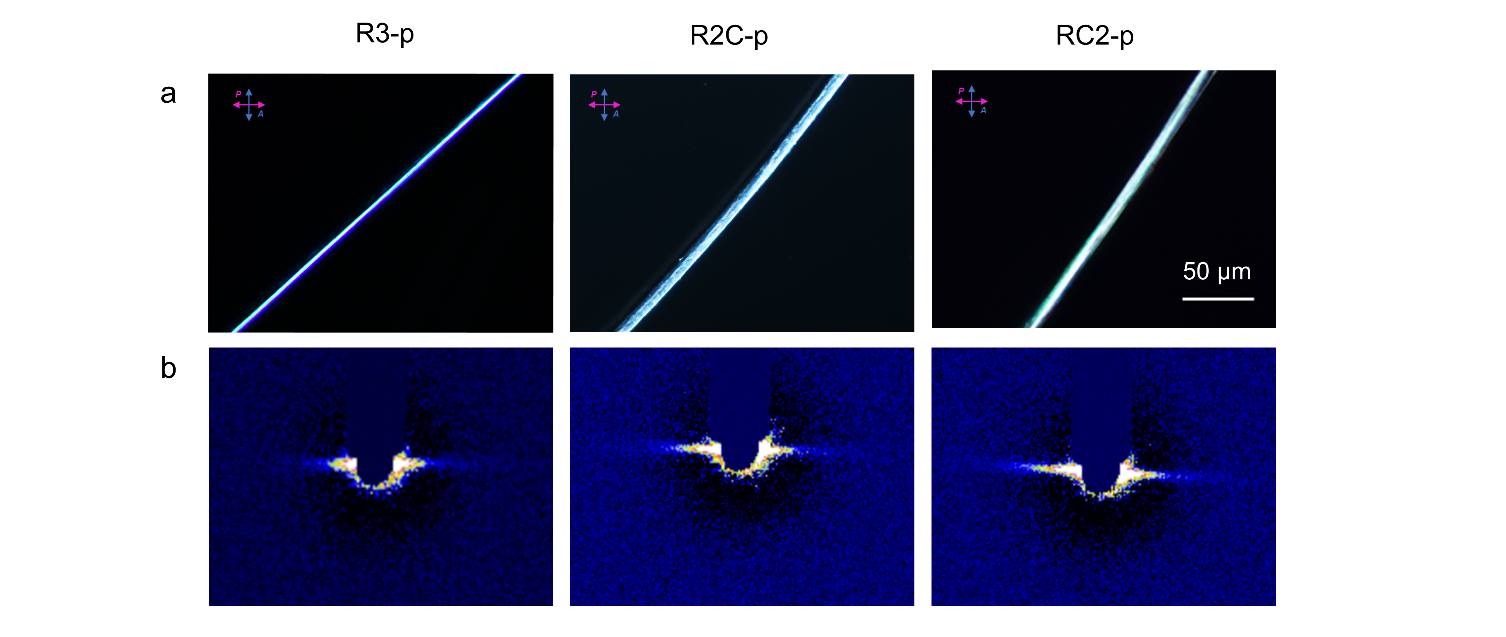


**Figure S7.** The POM (a) and SAXS (b) images of R3-p, R2C-p, RC2-p fibers.


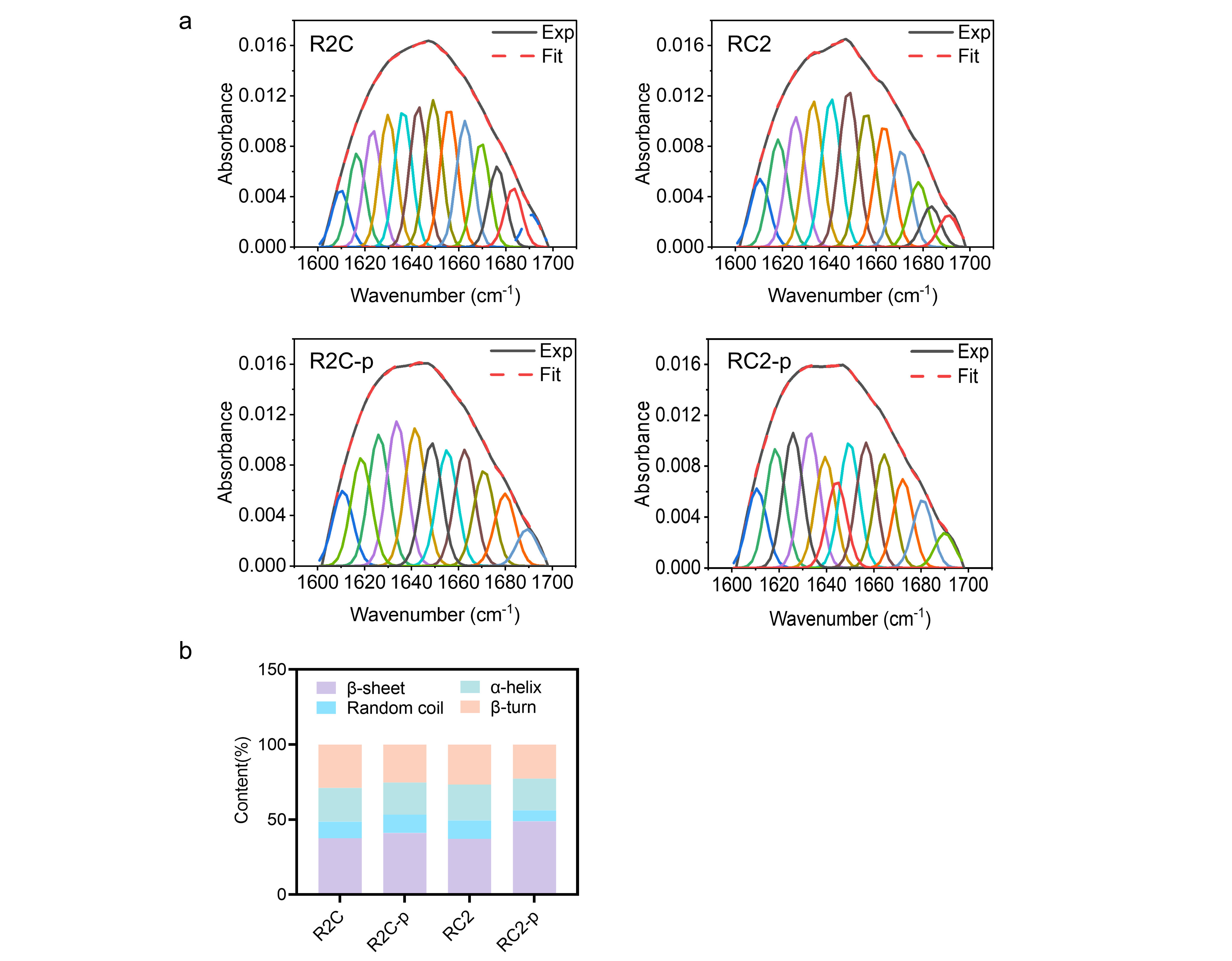


**Figure S8.** The FTIR analysis of as-spun and post-stretched R2C and RC2 protein fibers. (a) Peak fitting in FTIR amide I region of R2C, RC2 protein fibers before (up) and after (down) post-stretched. (b) Secondary structure contents in R2C, RC2 protein fibers with the calculation of FTIR.


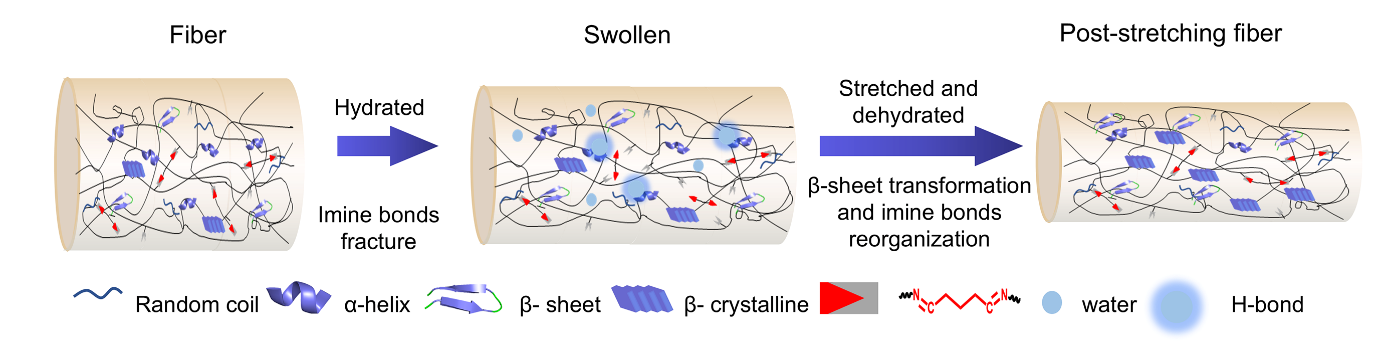


**Figure S9.** Schematics illustrating the underlying mechanism for the post-stretching of fibers.


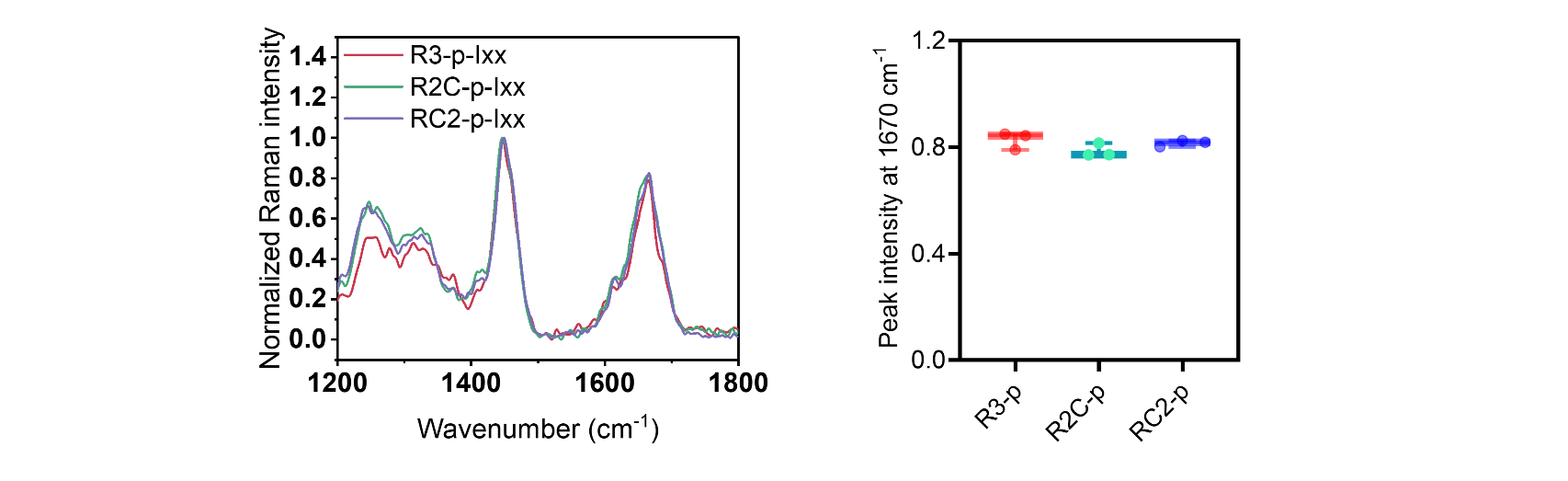


**Figure S10**. Polarized Raman spectra of different modular protein fibers on the Ixx direction and the peak intensity of three protein fibers at 1670 cm^-1^.


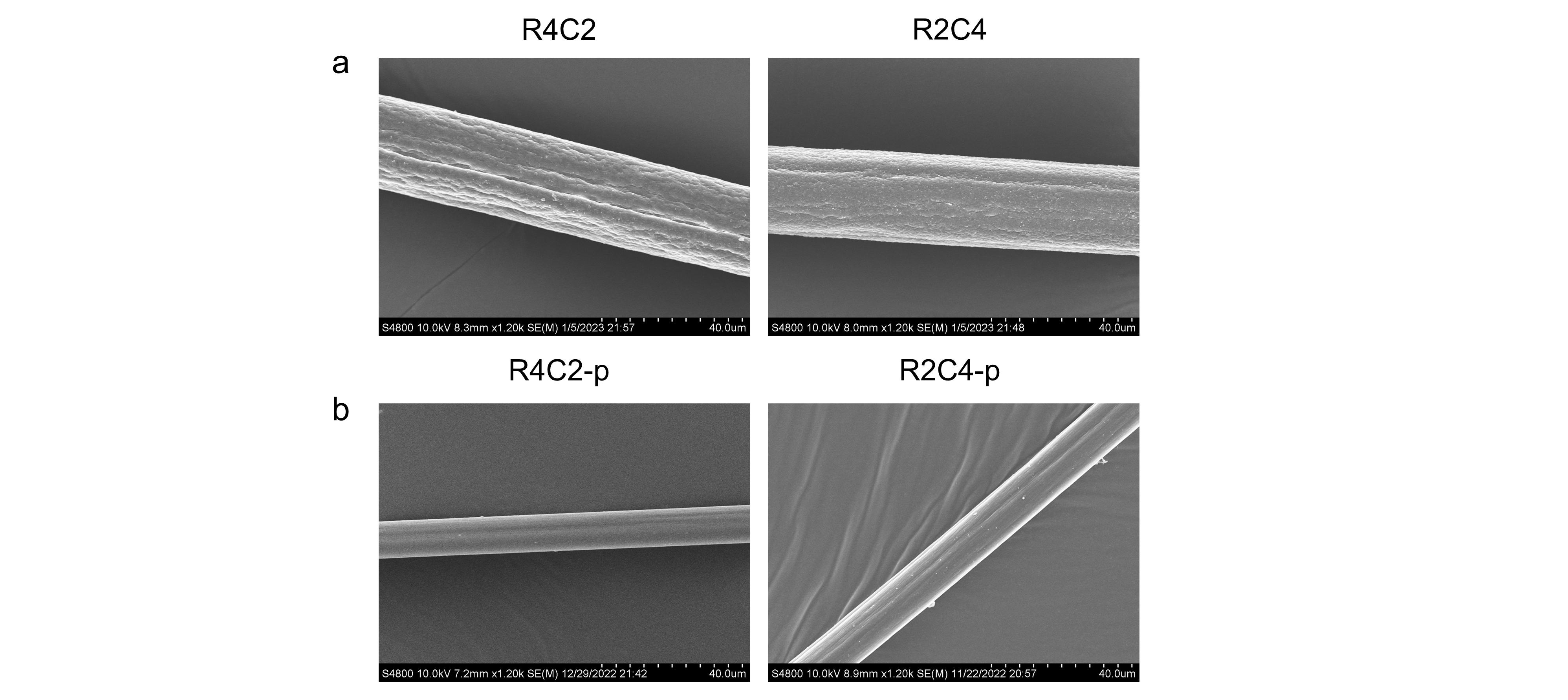


**Figure S11.** The SEM images of R4C2 and R2C4 protein fibers without (a) and with (b) post-stretching.


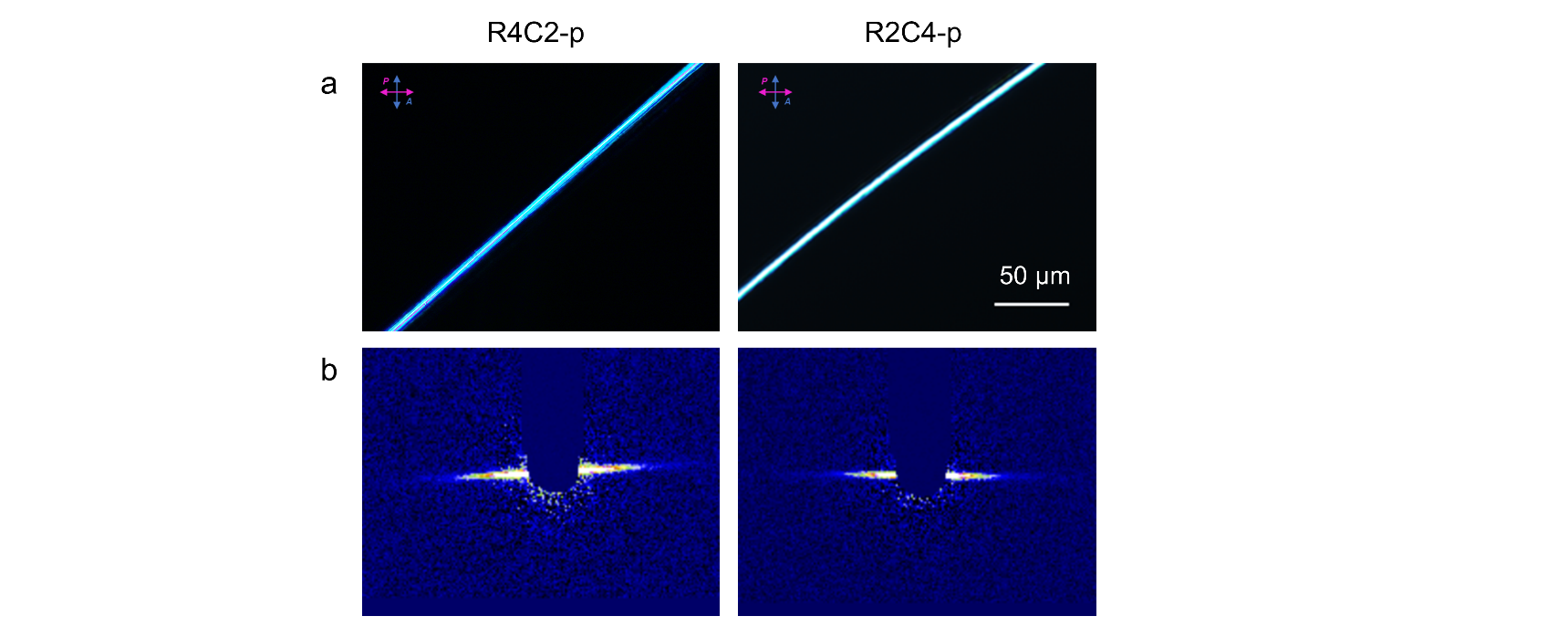


**Figure S12**. The POM (a) and SAXS (b) images of R4C2-p and R2C4-p fibers.


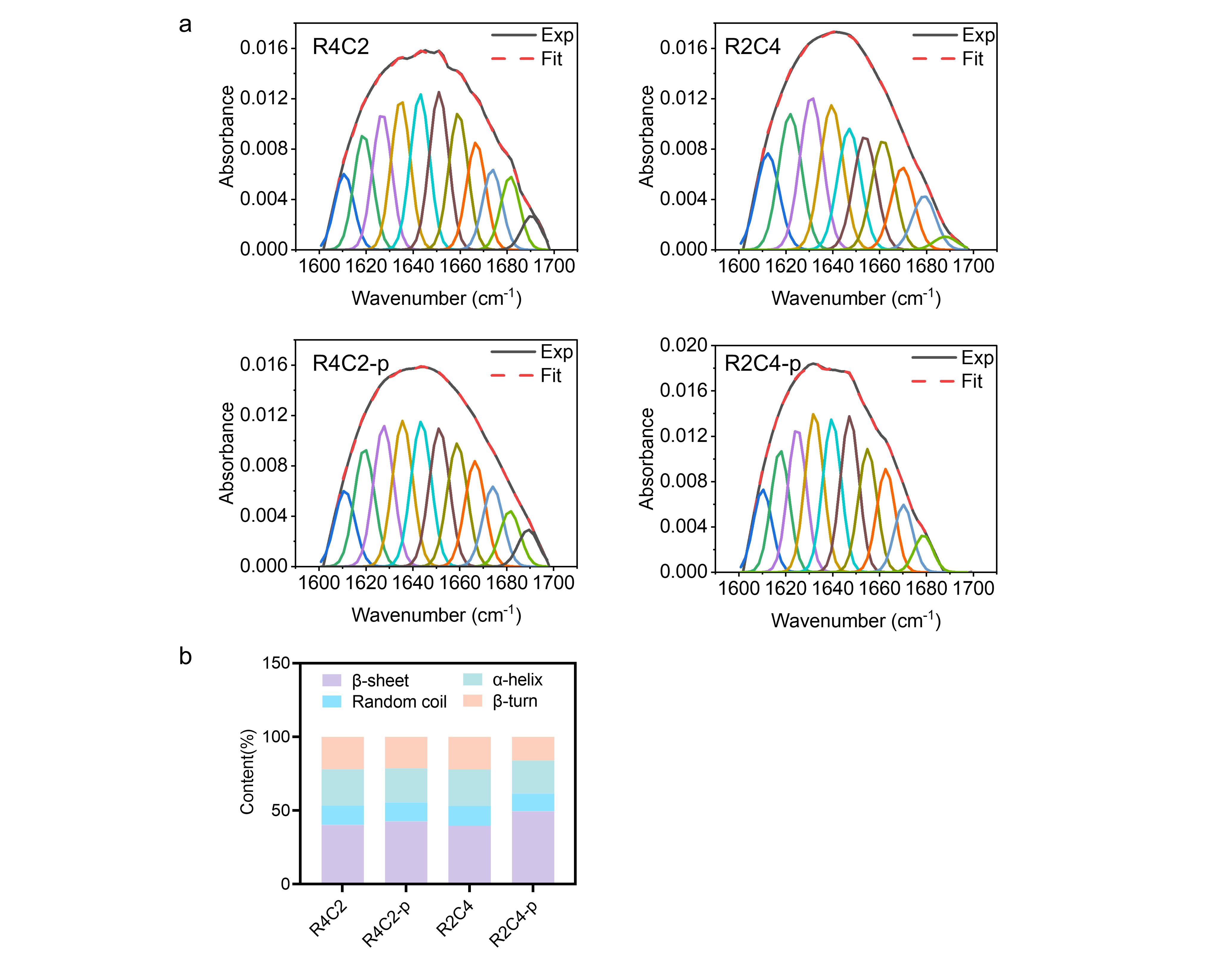


**Figure S13.** The FTIR analysis of as-spun and post-stretched R4C2 and R2C4 protein fibers. (a) Peak fitting in FTIR amide I region of R4C2, R2C4 protein fibers before (up) and after (down) post-stretching. (b) Secondary structure contents in R2C, RC2 protein fibers with the calculation of FTIR.


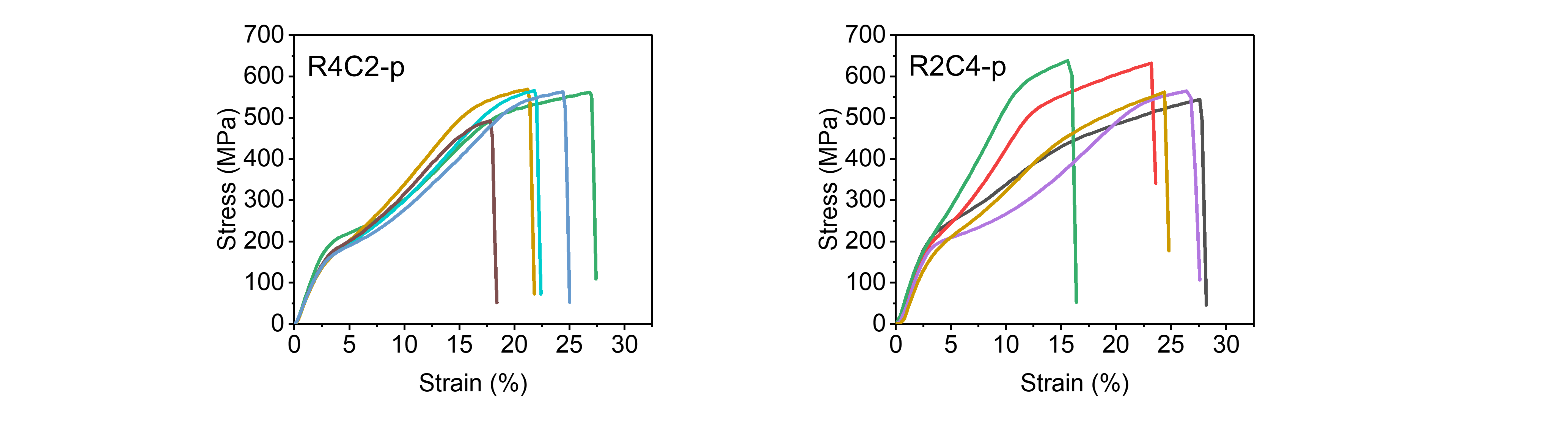


**Figure S14.** Typical stress-strain curves of post-stretched R4C2-p, R2C4-p protein fibers in this work.

**Table S5.** Summary of mechanical properties of post-stretched R4C2-p, R2C4-p protein fibers in this work. All data are presented as mean ± SD (n = 5).

|  | Diameter (μm) | Stress  (MPa) | Toughness (MJ/m^3^) | Modulus (GPa) | Fracture strain (%) |
| --- | --- | --- | --- | --- | --- |
| R4C2-p | 9.33±0.65 | 550.28±29.10 | 77.99±15.54 | 6.25±0.54 | 22.40±3.04 |
| R2C4-p | 9.06±0.60 | 588.25±39.30 | 90.60±13.98 | 7.49±0.33 | 23.44±4.21 |


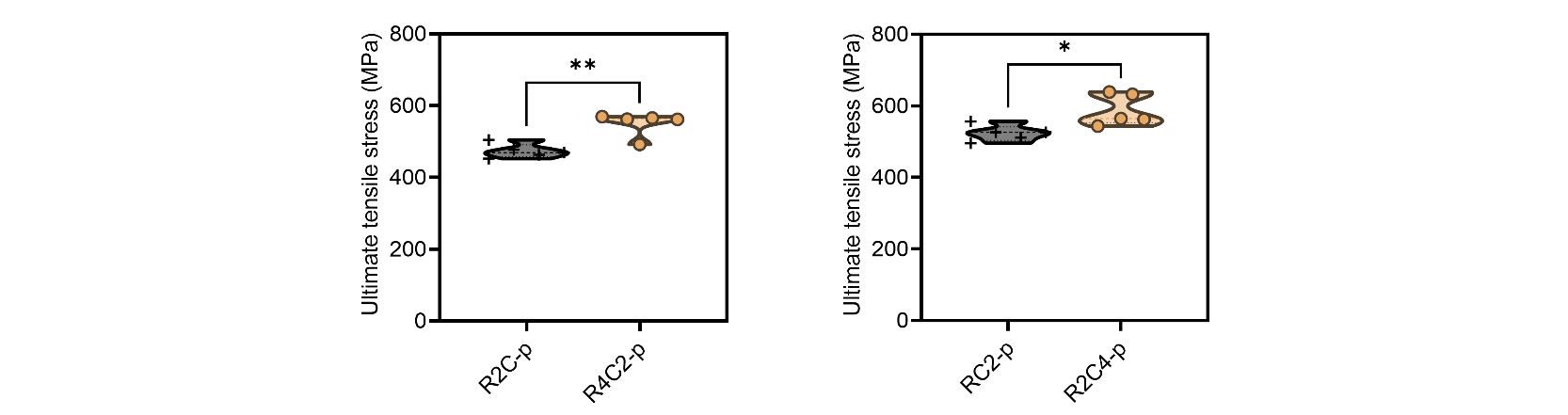


**Figure S15.** Comparison of mechanical properties of low- and medium-Mw protein fibers.


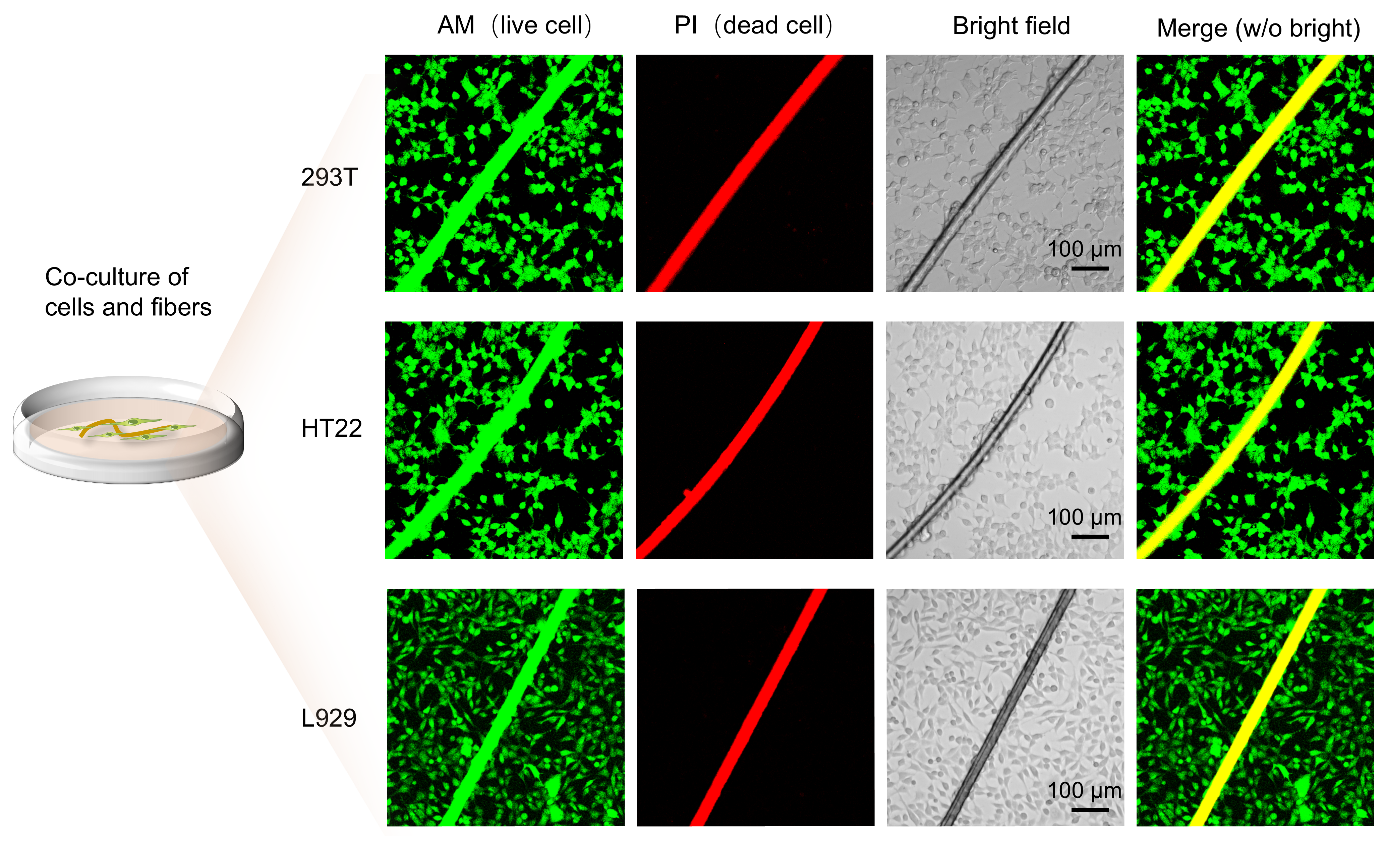


**Figure S16.** Cytotoxicity tests of the multi-module protein fibers. The fibers were cocultured with HEK293T, L929, or HT22 cells. The live cells are stained with AM and dead cells are stained with PI. Dead cells were barely observed and even living cells adhered on the fiber surfaces.

**References**

[1] D. Qin, M. Wang, W. Cheng, J. Chen, F. Wang, J. Sun, C. Ma, Y. Zhang, H. Zhang, H. Li, K. Liu, J. Li, *Angew. Chemie Int. Ed.* **2024**, *63*, e202400595.

[2] J. Su, B. Liu, H. He, C. Ma, B. Wei, M. Li, J. Li, F. Wang, J. Sun, K. Liu, H. Zhang, *Adv. Mater.* **2022**, *34*, 2200842.

[3] J. Li, Y. Zhu, H. Yu, B. Dai, Y.-S. Jun, F. Zhang, *ACS Nano* **2021**, *15*, 11843.
